# Perennial and seasonal contamination of maize by aflatoxins in eastern Kenya

**DOI:** 10.1186/s40550-018-0069-y

**Published:** 2018-08-31

**Authors:** Meshack Amos Obonyo, Elsie Nyangweso Salano

**Affiliations:** Department of Biochemistry and Molecular Biology, Egerton University, P. O. Box 536-20115, Egerton, Kenya

**Keywords:** Aspergillus, Risks, Aflatoxicosis, Maize, Seasonality

## Abstract

**Background:**

Aflatoxin contamination of grain is a major constraint to sustained quality cereal production. The causative fungi, *Aspergillus* species infect crops in the field and continue to do so post-harvest where they produce toxins in store. The current study aimed at establishing seasonal variation in levels and types of aflatoxins in maize from the Eastern region of Kenya- the hot-spot for aflatoxicosis. Maize kernels were collected from farmers’ households in May and December 2013 -2 months after long rain and short rain season respectively. The total aflatoxins were quantified using Enzyme-Linked Immunosorbent Assay (ELISA), while the toxin composition was determined using Thin-Layer Chromatography (TLC) and confirmed using High-Performance Liquid Chromatography (HPLC).

**Results:**

Generally, grain harvested after the long rains (May) had significantly (*p* = 0.019) lower aflatoxin levels and variation (5.68 ± 6.31 ppb, 100% Aflatoxin B1) than that of short rains (10.77 ± 10.14 ppb, 72% AFB_1_). Additionally, from the long and short rain seasons, the samples exceeding regulatory allowed limit (10 ppb) were 16 and 44% respectively.

**Conclusion:**

In Eastern Kenya, consumption of maize harvested in the long rain season presents a recurrent risk of exposure to low levels of AFB_1_; while consumption of maize harvested after the short rain season presents a risk of seasonal exposure to high levels and mixed type of toxins However, this long term risk of exposure to aflatoxins is poorly documented hence these findings necessitate mitigation measures because AFB_1–_ is a potent class 1 mutagenic toxin likely to cause liver cancer.

## Background

Over the last four decades, Kenya has become one of the leading countries in terms of incidence and severity of human exposure to aflatoxins (Mehl and Cotty 2010). The most severe episode in 2004 had 317 reported cases of which 125 were fatal (Probst et al. 2007). It is believed that the magnitude of exposure could be higher than reported for lack of robust monitoring systems. Consumption of aflatoxin contaminated grain has adverse health implications such as abdominal distensions, immune suppression (Cusumano et al. 1996), cancer, stunted growth in children (Gong et al. 2004) including death at high level of exposure (Probst et al. 2011). The majority of the fatalities reported in Kenya have occurred in the lower Eastern region of the country, which lead to a widely accepted view that the region is the hot-spot of aflatoxicosis.

The factors underlying repeated aflatoxicosis episodes in the hot-spot area of Kenya are yet to be unravelled. However, previous studies attribute high residual mycotoxin levels to climatic changes while others link it to ongoing anthropogenic causes such as delayed harvesting, poor drying and storage conditions (Torres et al. 2014). Among the important climatic factors are rainfall and temperature which affect residual grain moisture content and density of the aflatoxigenic fungi (Cotty and Jaime-Garcia 2007; Milani 2013). There is evidence showing a positive correlation between aflatoxins level and climatic seasons. This has been documented in in Nepal where aflatoxins were higher in crops harvested during the dry season (Gautam et al. 2008) while in Sierra Leone, human exposure to aflatoxins and ochratoxins appeared higher in the dry season than the rainy one suggesting higher contamination (Jonsyn-Ellis 2001).

The mentioned studies support the hypothesis that aflatoxicosis is largely seasonal with an opportunity for prediction hence mitigation. Therefore, it’s imperative to determine the nature of risk in the hot-spot region for appropriate intervention. The current study sought to achieve two objectives (i) establish the variation of aflatoxins in maize by cropping season and (ii) determine the predominant type of toxin in each respective season. To the best of our knowledge, this information is lacking hence hampering long-term management efforts in the hot-spot region of Eastern Kenya.

## Methods

### Sample collection

The study site (Eastern Kenya) is semi-arid with an annual conditions as follows: rainfall 250–500 mm, RH 60–70% and temperature 23–34 °C (Freeman and Coe 2002). Samples were collected in May and December 2013 approximately 2 months after harvest following the long and short rain season of the year. An altitudinal transect Machakos 1° 31′ 0.0120″ S37° 16′ 0.0120″ E [1000–1600 **masl**] to Kitui 1° 22′ 1.0560″ S38° 0′ 37.9800″ E [400–1163 **masl**]) was selected from which sampling points were set every 5 km in the 100 Km road. At each sampling, farmers on both sides of the main road were randomly selected. A sample consisted of half a kilogram of shelled maize Kernels that was being used for immediate consumption. Samples were separately kept in brown Khaki bags, labelled and transported in a cool box to the laboratory, stored at 4 °C until analysis. During the second season, repeated sampling was undertaken in the same areas. In total, about 200 samples were collected in both seasons but were segregated depending on whether the farmer had harvested the maize from their farm or purchased during either of the seasons. All samples from farmers who had purchased maize were excluded from the current results. Due to poor harvest experienced during in 2013, most farmers had purchased maize for their daily use. Thus, only a total of 50 maize samples from both seasons were analysed further.

### Quantification of total aflatoxins

Maize Kernels were ground to fine powder using a mill grinder. To 20 g of the maize powder, 100 ml of extraction solvent (70% methanol) was added in a conical flask. The flask was covered with aluminium foil and vortexed for 2 min. The mixture was then allowed to settle then filtered (Whatman # 1). 10 ml of the filtrate was drawn for aflatoxin testing using a solid phase direct competitive Enzyme-Linked Immunosorbent Assay, following manufacturer’s instruction (HELICA Biosystems Inc). The absorbance was read at 450 nm in a microplate reader (ThermoScientific). A standard curve was generated using the absorbance and the known concentrations of the six standards (HELICA Biosystems Inc). The concentrations of the samples were interpolated from the standard curve using GraphPad Prism version 6.0.5.0.

### Identification of aflatoxin types

Standard procedure for performing and analysis of aflatoxins by TLC were followed as described by Jager et al. (2013). Briefly, 20 g of maize powder was added to a 500 ml flat-bottomed flask. Into it, a mixture of 25 g hyflosupercel, 200 ml chloroform, and 20 ml distilled water was added then covered and shaken on a mechanical shaker for 30 min. After filtration (Whatman # 1) the first 100 ml was collected and concentrated. Two-thirds of a column chromatography tube was filled with chloroform after which 5 g of Na_2_SO_4_ was added. 10 g of silica gel slurry was transferred into the column and the setup allowed to stand for 15 min and 15 g of Na_2_SO_4_. The chloroform was drained and extract was transferred into the silica gel column. Hexane (100 ml) was added then drained followed by 100 ml of diethyl. Alfatoxins were eluted using 100 ml of a chloroform-methanol mixture (ratio 145.5:4.5) and collected into a round-bottomed flask for examination under UV light at 360 nm while compared to the standards. Afterwards, the positive samples were taken for further HPLC analysis in order to confirm the identity of the individual toxins, at the National Public Health Research Laboratories.

### Data analysis

Prior to analysis, the “equality of variances” test was used to inform the choice of T-test procedure. The test revealed insufficient evidence of unequal variances (the Folded F statistic *F*′ = 2.58, with *p* = 0.0240) between the two seasons. Hence, a pooled T-test was used for comparison of the total aflatoxins in maize samples drawn from the long and short rainfall seasons. The data was analysed using R Software version (3.3.1). In addition to comparison of the seasons, the number of samples unfit for human consumption (above 10 ppb) as well as prevalence of the different aflatoxins was also determined.

## Results

### Quantification of total aflatoxins

In general, maize from the long rain season had significantly (*p* = 0.0191) lower (mean ± SD) aflatoxin levels (5.68 ± 6.31 ppb) than those from the short rain season (10.77 ± 10.14 ppb) (Table 1).

**Table 1:** Summary T-test statistics for comparison of the long and short rain season

Season	Sample(n)	Mean aflatoxin	Std Dev	Std Err
Long Rain	25	5.68	6.31	1.26
Short Rain	25	10.77	10.14	2.03
Diff (1–2)		−5.09	8.45	2.39

Means obtained after equality of variance and pooled T-test (DF, 48, t value − 2.13, p = 0.0191)

Additionally, samples unsuitable for human consumption (aflatoxin > 10 ppb) were 16% (long season) and 25% (short season) respectively.

### Identification of aflatoxin types

Four types of aflatoxins (B_1_, B_2_, G_1_ and G_2_) were reported with AFB_1_ in 100% of samples in the long rain season while the short season were mixed aflatoxins. In the short rains season, the levels of toxins in decreasing order were: AFB_1_ 72%, AFG_1_ 28%, AFB_2_ 8%, and the least was AFG_2_ 4%. Additionally, there were 12% of the samples with AFB_1_ and AFG_1_ appearing as mixed contamination (Table 2).

**Table 2:** Seasonal variation of total aflatoxins (ppb) and toxin type in maize harvested after the long and short rain cropping season

Long cropping season (Season 1) n = 25			Short cropping season (Season 2) n = 25		
Sample Code	Total Toxins (ppb)	Toxin Type	Sample Code	Total Toxins (ppb)	Toxin Type
1EM14	3.6	B1	2EM02	30.0	B1, G1
1EM15	3.6	B1	2EM03	1.4	B1
1EM18	3.0	B1	2EM04	8.0	B1
1EM19	3.1	B1	2EM05	2.5	B1
1EM20	6.0	B1	2EM06	12.7	B1
1EM21	2.2	B1	2EM07	15.4	B2
1EM23	2.2	B1	2EM09	2.7	G1
1EM24	21.3	B1	2EM11	2.2	B1
1EM25	2.2	B1	2EM12	15.2	B2
1EM26	13.3	B1	2EM22	1.2	B1
1EM27	4.5	B1	2EM30	3.2	B1
1EM28	2.4	B1	2EM34	2.9	B1, G1
1EM29	1.2	B1	2EM35	24.3	G1
1EM30	4.0	B1	2EM42	2.0	B1
1EM32	3.5	B1	2EM43	1.5	B1
1EM33	18.4	B1	2EM51	1.6	B1
1EM35	1.9	B1	2EM52	3.2	B1
1EM36	5.1	B1	2EM54	19.2	B1
1EM41	2.6	B1	2EM55	10.0	G1
1EM42	23.7	B1	2EM61	2.6	B1
1EM45	2.8	B1	2EM63	27.0	G1
1EM46	4.3	B1	2EM66	19.8	B1
1EM47	3.0	B1	2EM86	25.6	B1, G1
1EM48	2.8	B1	2EM87	28.9	G2
1EM49	1.3	B1	2EM92	6.2	B1
Mean ± SD	5.68 ± 6.31			10.77 ± 10.14	

Mean ± SD aflatoxin concentration independent t-tests show significant difference (*p* = 0.0191) between the long and short rain season

## Discussion

Aflatoxin contamination of grain is an impediment to quality food production and trade across the globe (Kebede et al. 2012). In Kenya, it’s a life-threatening phenomenon because aflatoxicosis occurs with concomitant human fatalities. The current study was designed to explain the observed high levels of aflatoxins in the region by separating contamination of grain that is due to grain importation into the hot-spot region from that which is due to local production. The findings show that from 25% of grain consumed in 2013 in the sampled region, there is a twofold increase in aflatoxins recorded in maize consumed during the short rain season compared to the long rain season. Recently, in western Kenya-a major maize and peanut producing region of the country, the incidence of three maize ear rots (*Fusarium, Penicillium*, and *Aspergillus*) and their corresponding toxins were severe during the long rain season than the short one (Juti 2017). While the recent findings could be explained by the climatic differences between the two agro-ecological zones (Mugo et al. 2016), the studies attest to seasonal variation in level toxins in food material.

The current findings form an important basis for planning intervention against dietary exposure to aflatoxins since the rainfall pattern in major parts of the country is bimodal characterised by long and short rainy seasons interspersed with brief dry spells (Mugo et al. 2016). A seasonal assessment of aflatoxin residues in food is an important indicator of aflatoxicosis risk especially in view of climatic changes precipitating hot and dry spells associated with increased aflatoxin contamination (Cotty and Jaime-Garcia 2007; Kebede et al. 2012; Smith et al. 2016).

Adverse effects of consumption of aflatoxin contaminated food is linked to a large population of children experiencing poor and stunted growth in Benin (Gong et al. 2004) and in Eastern Kenya (Hoffmann et al. 2015). We hypothesise that the reported stunting effects in Eastern Kenya hot-spot may be attributable to perennial consumption AFB_1_, −a potent class 1 mutagenic and teratogenic toxin (Birch and Parker 2012; FAO, United Nations 2017). The findings of the current study correspond with the period when the most severe case of aflatoxicosis occurred (between May and June 2004) in Eastern Kenya after the long rain season (Anonymous 2004). This could be explained by the predominant AFB_1_ observed in the current study and also the same the same period. It is likely that in 2004, AFB_1_ levels were at a record high and resulted in human fatalities (Probst et al. 2007).

The seasonal variations in levels of toxins is likely the result of variations in the fungal community structure between cropping seasons. That could explain why AFB_1_ is predominant in the long rain season while there mixed toxins in the short season (Salano et al. 2016). There is also possibility of complex fungal interactions at their specific niche leading to differential toxin production (Huang et al. 2011; Okoth et al. 2018). The possibility of intra – and interspecific interaction between members of a fungi genus remains a confounding phenomenon but deserves attention. Therefore, it remains unclear why AFB_2_ and AFG_1_ were present in the second season only whereas the fungi known to produce them were present in isolates from both seasons (Salano et al. 2016).

## Conclusions

The seasonal pattern of aflatoxin contamination of the staple food –maize in Eastern Kenya, presents an opportunity for interventions such as biological control to reduce aflatoxins especially in the long rain season where AFB1 is uniformly produced (Bandyopadhyay et al. 2007; Cotty et al. 2008; Bandyopadhyay et al. 2016). While there is a lack of feasible intervention, the risk of chronic exposure to AFB1 remains high and poorly documented in both cropping seasons. Hence the findings of this study necessitate mitigation measures because AFB_1–_ is a potent class 1 mutagenic toxin likely to cause liver cancer.

## Data Availability

“Data sharing not applicable to this article as no datasets were generated or analysed during the current study.”

## Abbreviations

AFB_1_: Aflatoxin B1;
AFB_2_: Aflatoxin B2;
AFG_1_: Aflatoxin G1;
AFG_2_: Aflatoxin G2;
ELISA: Enzyme-linked immunosorbent assay;
FAO: Food and agriculture organization;
HPLC: High-performance liquid chromatography;
masl: meters above sea level;
RH: Relative humidity;
TLC: Thin-layer chromatography
